# The Relationship among Bowel [18]F-FDG PET Uptake, Pathological Complete Response, and Eating Habits in Breast Cancer Patients Undergoing Neoadjuvant Chemotherapy

**DOI:** 10.3390/nu15010211

**Published:** 2023-01-01

**Authors:** Paola Tiberio, Lidija Antunovic, Mariangela Gaudio, Alessandro Viganò, Manuela Pastore, Chiara Miggiano, Flavia Jacobs, Chiara Benvenuti, Elisabetta Farina, Arturo Chiti, Armando Santoro, Rita De Sanctis

**Affiliations:** 1 Medical Oncology and Hematology Unit, IRCCS Humanitas Research Hospital, Rozzano, 20089 Milan, Italy; 2 Nuclear Medicine Unit, IRCCS Humanitas Research Hospital, Rozzano, 20089 Milan, Italy; 3 Department of Biomedical Sciences, Humanitas University, Pieve Emanuele, 20072 Milan, Italy; 4 IRCCS Fondazione Don Carlo Gnocchi, 20148 Milan, Italy

**Keywords:** breast cancer, neoadjuvant chemotherapy, bowel [18]F-FDG PET uptake, nutrition, bowel inflammation, pathologic complete response

## Abstract

Recently, the impact of patients’ eating habits on both breast cancer (BC) management and inflammation have been proven. Here, we investigated whether inflammatory habits could correlate with baseline bowel [18]F-fluorodeoxyglucose (FDG) uptake and the latter, in turn, with pathological Complete Response (pCR) to neoadjuvant chemotherapy (NAC). We included stage I–III BC undergoing standard NAC at IRCCS Humanitas Research Hospital, Italy. Patients fulfilled a survey concerning eating/lifestyle behaviors and performed a staging [18]F-FDG positrone emission tomography/computed tomography (PET/CT). In the absence of data on the effects of individual foods, we aggregated drink and food intake for their known inflammatory properties. Data were recorded for 82 women (median age, 48). We found positive correlations between colon mean standardized uptake value (SUV_mean_) and pro-inflammatory drinks (alcohol and spirits; r = +0.33, *p* < 0.01) and foods (red and cured meats; r = +0.25, *p* = 0.04), and a significant negative correlation between rectum SUV_mean_ and anti-inflammatory foods (fruits and vegetables; r = −0.23, *p* = 0.04). Furthermore, colon SUV_mean_ was significantly lower in patients with pCR compared to non pCR (*p* = 0.02). Our study showed, for the first time, that patients’ eating habits affected bowel [18]F-FDG uptake and that colon SUV_mean_ correlated with pCR, suggesting that PET scan could be an instrument for identifying patients presenting unhealthy behaviors.

## 1. Introduction

Breast cancer (BC) is the most common neoplasm and the primary cause of cancer death in women worldwide. Despite its high incidence, there is a progressive decrease in cancer mortality and a consequently ever increasing number of cancer survivors [1]. However, many parameters could influence tumor development and survivors’ quality of life. Among modifiable risk factors, eating habits, body weight, and lifestyle behaviors have been deeply investigated [2,3,4,5,6,7,8]. Increasing evidence suggested that diet plays an important role in cancer development, progression, and prevention, including BC [9,10]. Several studies showed that healthy diet and exercise might improve overall survival and quality of life after BC diagnosis by reducing chemotherapy side effects, limiting comorbidities, and enhancing therapeutic efficacy [11,12,13,14,15].

[18]F-fluorodeoxyglucose (FDG) positrone emission tomography/computed tomography (PET/CT) is a functional imaging technique with extensive use in oncology for staging, as well as for assessment of cancer relapse and response to therapy [16,17,18], but also in different non-oncological diseases related to infection and inflammation. Interestingly, recent studies have also shown that [18]F-FDG PET/CT may be useful for detecting benign bowel inflammatory activity [19,20,21].

Despite the large number of studies investigating the association between diet and cancer risk, as well as exploring changes in eating habits in cancer survivors, to date there are no investigation regarding the possible influence of baseline dietary patterns on response to therapy in BC. In this study, we aimed to investigate whether, in BC patients, diet could correlate with bowel [18]F-FDG uptake and the latter, in turn, with pathological Complete Response (pCR) to standard neoadjuvant chemotherapy (NAC).

## 2. Materials and Methods

### 2.1. Study Design and Participants

We performed a prospective mono-centric longitudinal observational proof-of-principle study, enrolling women who underwent standard NAC for BC at IRCCS Humanitas Research Hospital in Rozzano, Italy.

The inclusion criteria were:

(a) willingness to participate to the study;
(b) age ≥ 18 years old;
(c) female gender;
(d) histopathologically confirmed diagnosis of BC;
(e) clinical stage T1c-T4, N0-N3, M0 at presentation;
(f) Eastern Cooperative Oncology Group (ECOG) Performance Status 0–1;
(g) baseline left ventricular ejection fraction ≥ 55%;
(h) adequate hematologic, liver and hepatic function;
(i) ability to give informed consent according to International Conference on Harmonization /European Union Good Clinical Practice, and national/local regulation.

The exclusion criteria were:

- inability to respond to survey;
- prior history of invasive BC;
- stage IV BC;
- prior systemic therapy for BC;
- previous therapy with anthracyclines/taxanes for any malignancy;
- use of immunomodulatory agents at the time of enrolment/during the previous 2 months;
- use of antibiotics at the time of enrolment/during the previous month;
- history of other malignancy within 5 years prior to the enrolment;
- pregnancy/breastfeeding/intention of becoming pregnant during the study.

At baseline, participants performed a whole-body staging [18]F-FDG PET/CT scan according to the recommendations of the European Association of Nuclear Medicine (EANM) guidelines [22]. NAC was administered according to international and national clinical guidelines after a multidisciplinary discussion on each single case. NAC regimens included anthracycline-based chemotherapy followed by (i) weekly carboplatin and paclitaxel in case of triple-negative BC, (ii) docetaxel and trastuzumab in case of human epidermal growth factor receptor 2 (HER2) positive BC, or (iii) docetaxel alone in case of luminal-like disease. Data on pathological response were collected and regular follow-up of the patients was performed. A flowchart of the recruitment and follow-up process is reported in Supplementary Materials Figure S1. The study was approved by the IRCCS Humanitas Research Hospital Ethics Committee (Protocol identifying number ONC/OSS-02/2019). All patients signed the informed consent form in accordance with the Declaration of Helsinki.

### 2.2. Survey Design

At baseline, before NAC therapy, all enrolled women fulfilled a survey developed by our dietitian nutritionist following national and international guidelines, which concerned their eating habits and the frequency of their physical activity. Specifically, patients have been asked to describe their diet as omnivorous/varied, vegetarian, or vegan and to report weekly frequencies of the consumption of 16 food items: milk, dairy products, alcoholic drinks, spirits, white meat, read meat, eggs, fruits, vegetables, fish, pulses, cereals, cured meat, salty snacks, sweet snacks/drinks, and nuts. The survey also investigated whether BC patients performed exercise regularly (stated as weekly frequency and type of exercise; a copy of the survey is reported in Supplementary Materials Figure S2). The questionnaire was self-administered, easy to understand, and provided semi-quantitative data. Food consumption frequencies of BC patients were analyzed in comparison with an ideal healthy diet (2000 kcal/die) [23] and results were reported as “more”, “correct”, or “less”. When no responses were provided, we indicated it as “missing data”.

### 2.3. [18]F-FDG PET/CT Acquisition Protocol

Fasting for at least 6 h prior radiopharmaceutical injection and rest (restrained from excess physical activity and talking) were required as preparation for [18]F-FDG PET/CT imaging. Prior to radiotracer injection, blood glucose level measurements were obtained if serum glucose concentration was lower than 200 mg/dL, an intravenous injection of ~6 Megabecquerel (Mbq)/kg of [18]F-FDG was performed. Post-injection, a one-hour interlude was mandatory for all participants.

Subsequently, each patient was scanned using one of two integrated PET-CT scanners: a Siemens Biograph LS 6 scanner (Siemens, Munich, Germany), or a GE Discovery PET-CT 690 (General Electric Healthcare, Waukesha, WI, USA). After attenuation correction, images were reconstructed obtaining axial, sagittal, coronal CT, PET, and PET/CT fused images.

On axial images, an experienced nuclear medicine physician designed regions (ROI) to extract values of semi-quantitative parameters of radiotracer mean standardized uptake value (SUV_mean_). Two ROIs were positioned on the area of highest uptake respectively in the rectum-sigmoid district and in the remaining part of the colon.

### 2.4. Statistical Analysis

Results were presented as means ± standard deviations, medians and ranges, or percentages of the total. Parametric or non-parametric tests were used according to the data mean distribution.

Correlation analysis was performed by using Pearson’s coefficient between SUV_mean_ of colon and rectum on one side, and dietary habits, frequency of physical activity, body mass index (BMI), and smoking habit on the other. Concerning eating habits, in the absence of data on the effects of individual foods, weekly intake of well-known pro- and anti-inflammatory drinks or foods were aggregated. Specifically, weekly frequency of consumption of alcoholic drinks and spirits were added together and referred to as “pro-inflammatory drinks”. In the same way, we putted together intakes of red and cured meats (“pro-inflammatory foods”) and of fruits and vegetable (“anti-inflammatory foods”). We performed a multiple comparison correction by False Discovery Rate (FDR).

Differences between values of bowel SUV_mean_ in patients obtaining pCR and non pCR were tested using a two-sided *t*-test. Analyses were run separately for the SUV_mean_ values of the rectum and of the colon.

Moreover, multivariate approach was carried out using Discriminant Function Analysis (DFA), which is a powerful tool to build associative models with categorical outcome of interest, as in this case (pCR versus non pCR). A deeper description of DFA is provided elsewhere [24,25,26,27]. In brief, DFA estimates the linear combination of selected covariates able to split single cases into groups according to an outcome of interest. DFA provides a model in which the variables associated to the outcome are listed according to their weight in decreasing order. Significance level was set at <0.05 after proper correction. Statistics were performed with STATISTICA, version 7, StatSoft, OK, USA.

## 3. Results

### 3.1. Patients’ and Tumors’ Characteristics

Demographic details of the 82 patients enrolled in the study are depicted in Table 1 and Table 2. Both average and median age were 48 years (range, 25–72 years). Focusing on BC risk factors, 18.3% of the population were smokers, whereas 64.6% never smoked and the remaining 17.1% stopped smoking before BC diagnosis (median time from quitting smoking, 10 years). Most women were premenopausal (57.3%), and the average BMI was 23.68 (range, 16.18–35.82). An amount of 61% of women had a normal BMI, 4.9% were underweight, and 30.5% were overweight, whereas only 3.7% were obese. Eleven patients had a gastrointestinal comorbidity and only one had insulin-dependent diabetes mellitus. We conducted an analysis of variance ANOVA to test whether comorbidities could influence levels of bowel [18]F-FDG uptake with no significant differences (*p* = 0.50). All patients were diagnosed with stage IA–IIIB BC and most tumors were high grade (43 patients had G3 tumor). Furthermore, 45 women were diagnosed with a HER2 + BC, 29 had a triple-negative breast cancer (TNBC), and the remaining presented luminal-like tumors (one Luminal A and seven Luminal B). At the end of NAC, 37 patients reached pCR, whereas 45 did not.

### 3.2. Eating Habits

**Figure 1:**
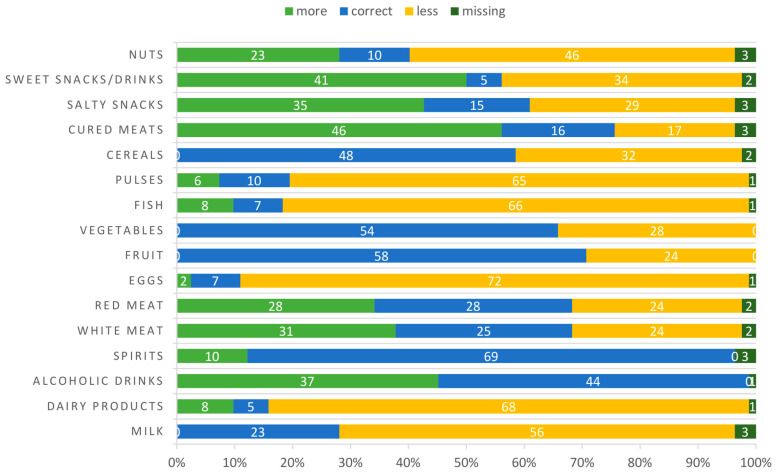
Distribution of patients’ food consumption frequencies compared to an ideal healthy diet. “Missing” indicate that no responses were provided. White numbers in the bar represent number of patients.

Almost all enrolled women were omnivorous, two patients followed a vegetarian diet, whereas only one was vegan. In Figure 1 and Table 3, eating habits of participants alone and compared to an ideal healthy diet [23] are illustrated, respectively. Most women showed a correct intake of alcohol and spirits (weekly frequency equal to 0), as well as fruits, vegetables, and cereals. However, weekly consumption of milk, diary supplements, eggs, fish, pulses, and nuts was under the healthy recommended level [23]. On the contrary, there was an overconsumption of cured meats and snacks. Focusing on the strongest dietary recommendation for cancer patients (i.e., a correct intake of white meat, fruits and vegetables, fish, and pulses or cereals) [28], we noticed that only 3.7% of patients followed a healthy diet before BC diagnosis. Interestingly, 52.4% of patients declared that they usually exercise regularly (Table 4).

### 3.3. Correlation between Eating and Exercise Habits and Bowel [18]F-FDG Uptake

We then investigated whether increased levels of bowel [18]F-FDG uptake observed at the staging PET scan could be influenced by patients’ lifestyle, considering both eating and exercise habits. After FDR correction, we found a positive correlation between baseline colon SUV_mean_ and pro-inflammatory drinks (r = +0.33, *p* < 0.01) and foods (r = +0.25, *p* = 0.04). A significant negative correlation was also observed between baseline rectum SUV_mean_ and anti-inflammatory foods (r = −0.23, *p* = 0.04) (Table 5). No statistically significant associations were seen with BMI, smoking habits, or physical activity.

### 3.4. Association between Bowel [18]F-FDG Uptake and Response to Therapy

**Figure 2:**
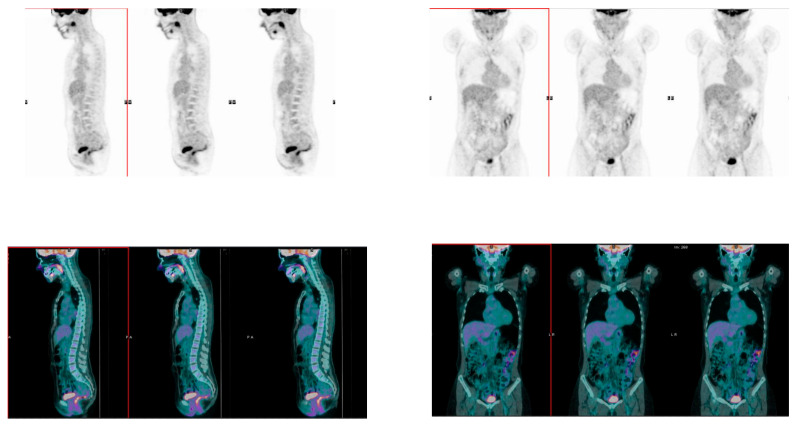
Sagittal (left panel) and coronal (right panel) images of whole-body [18]F-FDG PET/CT scan. PET (upper panels) and fused PET/CT (lower panels) images, showing faint diffuse [18]F-FDG colon and rectum uptake in a BC patient who reached pCR. FDG, fluorodeoxyglucose; PET/CT, positron emission tomography/computed tomography; BC, breast cancer; pCR, pathological Complete Response; PA: posterior-anterior, ; LR: left-right.

**Figure 3:**
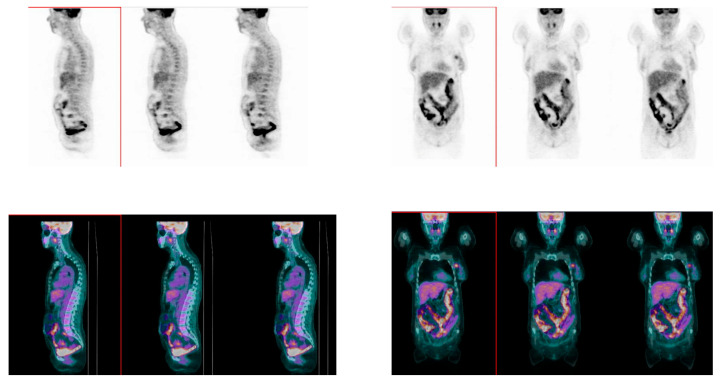
Sagittal (left panel) and coronal (right panel) images of whole-body [18]F-FDG PET/CT scan. PET (upper panels) and fused PET/CT (lower panels) images, showing diffuse nonhomogeneous [18]F-FDG uptake in colon and rectum in a BC patient who did not reach pCR.

**Figure 4:**
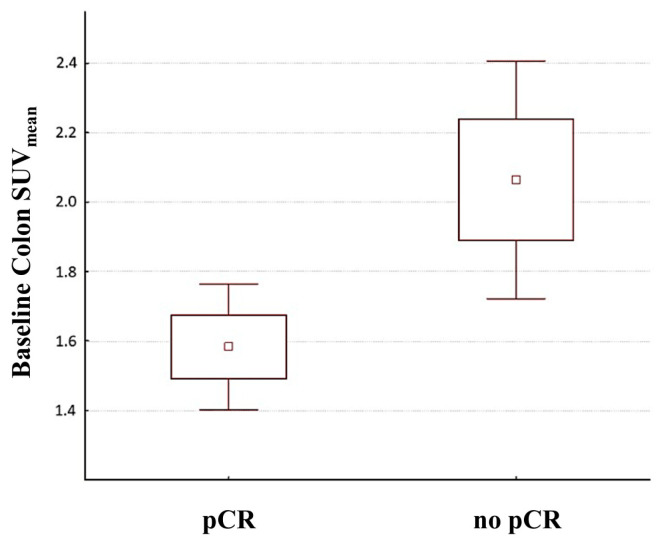
Distribution of baseline colon SUV_mean_ values in patients who subsequently experienced pCR after NAC compared to no pCR patients. The mean value for each group is indicated by a central square. Boxes indicate the Standard Error. Whiskers represent 1.96 × Standard Error. NAC, neoadjuvant chemotherapy; SUV_mean_, mean standardized uptake value.

At baseline, rectum SUV_mean_ did not differ between patients who experienced a pCR (1.99 ± 0.59) and patients who did not (2.13 ± 1.11) (*p* = 0.48). On the other hand, colon SUV_mean_ was significantly lower in patients who experienced pCR after NAC (1.58 ± 0.56; Figure 2) compared with non pCR patients (2.05 ± 1.17; Figure 3) (*p* = 0.02; Figure 4).

The multivariate approach confirmed results from the univariate analysis (F(6.67) = 2.49; *p* < 0.03). DFA model pinpointed the factors that were significantly associated to pCR: colon SUV_mean_, cured meats, rectum SUV_mean_, fruits, alcoholic drinks, and red meat. Together, these factors could explain up to 98.1% of the variance, and the first two factors (i.e., colon SUV_mean_ and cured meat) explained by themselves more than 90% of the variance (see Supplementary Materials Table S1).

## 4. Discussion

Currently, it is well established that maintaining a healthy weight, being physically active, and following healthy eating patterns can reduce cancer risk and increase patients’ outcome and quality of life [4,5,9,10]. Besides their impact on cancer management, dietary components have profound effects on inflammation. On one hand, human diet is a highly complex mixture of chemical compounds which make it difficult to clearly predict the final result of their overall effects. On the other hand, several dietary components have been demonstrated to modify cancer risk by modulating systemic inflammation. Some nutrients like omega-3 fatty acids and fiber can reduce inflammation, while others like refined carbohydrates, cholesterol, and saturated fatty acids have pro-inflammatory activities [29,30,31,32,33,34,35]. However, to our knowledge, no data has emerged, until now, concerning the influence of pro-inflammatory habits on response to NAC in BC patients. Here, we investigated this association, taking advantage of bowel [18]F-FDG uptake measured before NAC as a parameter for detecting bowel inflammation induced by unhealthy habits.

In agreement with previous data [28], in our study population at the time of diagnosis, the percentage of women following all recommendations included in an ideal healthy diet was very low (3.7%). Nevertheless, we noticed that most BC patients had a correct intake of fruits, vegetables, and cereals, did not drink alcohol or spirits, had an appropriate BMI, and regularly performed physical activity, just as suggested for cancer prevention and management [4,9]. On the other hand, in our cohort, few women adhered to recommendations concerning red and processed meat, which were classified by World Health Organization’s International Agency for Research on Cancer as probably carcinogen and carcinogen, respectively [9]. Indeed, more than one third of the patients overemployed their consumption and about half of them abused cured meat.

Besides their effects on cancer, unhealthy habits may have an impact on bowel inflammation [29,30,31,32,33,34,36]. Thus, we assessed whether increased levels of bowel [18]F-FDG uptake correlated with well-known pro-inflammatory foods could be a marker of bowel inflammation. Drinks and foods were aggregated considering their well-known pro- or anti-inflammatory activities, since no data on the effects of individual foods were available. We observed that colon [18]F-FDG uptake positively correlated with the consumption of pro-inflammatory drinks and foods, whereas rectum [18]F-FDG uptake was inversely associated with anti-inflammatory food intake. Interestingly, colon SUV_mean_ showed a negative correlation trend with anti-inflammatory foods (*p* = 0.05). These findings reflect, at least in part, literature results. In fact, it has been proven that a diet rich in fats, processed meats, and sweet/salty snacks increased serum inflammatory markers [37]. Contrary, a decrease in inflammatory factors was associated with dietary patterns rich in fruits and vegetables [31,32,33,34]. Thus, we can speculate that bowel [18]F-FDG uptake may be affected by both pro- and anti-inflammatory foods and that we may use PET scan as an instrument for the identification of patients with increased levels of [18]F-FDG uptake suggestive of bowel inflammation. Furthermore, it could be useful to discuss the possible mechanisms influencing the absorption of [18]F-FDG in the small and large bowel. A low-carbohydrate, high-fat diet in the hours immediately before the PET examination reduced the [18]F-FDG uptake in the descending colon and small bowel when compared to a routine diet [38], possibly exerting its effect acting on the Randle cycle as previously demonstrated [39]. However, this condition should have marginally influenced our case since all patients were on the same dietary limitations (low-carbohydrate diet since lunch on the day preceding the PET scan). Surprisingly, we have shown here, for the first time, that colon mean [18]F-FDG uptake was also inversely correlated with pCR, thus suggesting a role for colonic inflammation and possibly its causative unhealthy foods and beverages in NAC response. Furthermore, since pCR is considered a surrogate endpoint for long-term outcome [40], we could speculate that unhealthy foods that trigger colonic inflammation may have an impact on long-term outcome in BC patients. These results have also been confirmed by the multivariate analysis carried out with DFA that highlighted that pCR was more strongly influenced by colon [18]F-FDG uptake and cured meat; these were the variables that most influenced the discrimination between pCR and not-pCR cases.

The influence of dietary patterns is widely recognized in inflammatory bowel disease. An unhealthy diet, rich in processed meat and low in fibers, has been associated with alterations in the gut microbiome and barrier function of the colonic epithelium [41]. In particular, some evidence indicated that fiber is more effective than the Mediterranean diet on influencing the gut microbiota composition [42,43,44,45,46]. In vivo studies have shown that disruption of the healthy gut microbiota has direct effects on the immune system by triggering a pro-inflammatory environment controlled by specific subpopulations of the immune system (e.g., natural killer cells) [47]. Therefore, a healthy diet including high-fiber foods, such as fruits, vegetables, and whole grains, could effectively reduce the risk of several metabolic diseases, including colorectal and breast cancer [48,49,50]. In addition, previous studies have demonstrated an association between gut microbiota and physiologic bowel [18]F-FDG activity both in healthy subjects and BC patients [51,52]. In healthy subjects, different levels of bowel uptake were associated with specific microbial taxa, thus suggesting that an increased [18]F-FDG uptake might be caused by an increment in intestinal permeability and might reflect impaired intestinal barrier function [51]. On the other hand, Yoon and colleagues found that changes in intestinal bacteria abundance in BC patients were associated with physiological intestinal [18]F-FDG and that the latter was associated with pro-inflammatory Tumor Necrosis Factor-α, thus further supporting the link between mucosal inflammation and physiologic intestinal [18]F-FDG uptake [52].

In the present study, no association was observed between the rectum SUV_mean_ and the pCR. However, compared to the rectum, the colon performs most of the large bowel functions. In addition to systemic immune control and microbiota function, colon physiology is determined by the role of the various epithelial cells that form its mucosa and are responsible for water and electrolyte absorption [53]. In addition, probiotic bacteria in the colon flora, such as *Lactobacillus* and *Bifidobacterium*, regulate micronutrient levels such as vitamins (e.g., folate-producing strains) and exert an immunomodulatory effect [54]. Therefore, the impact of inflammation at the colon level is far more compelling, due to its multiple functions that could interfere with response to NAC, than the possible inflammatory alterations of the rectum, which is primarily responsible as a reservoir for fecal content [55].

Different studies have investigated the effect of exercise in counteracting inflammation [56], and in obtaining benefits for patients with cancer [4,12]. However, in this population, no statistical significance has been reached between physical activity and bowel [18]F-FDG uptake, despite correlation analyses suggested an inverse association. This could be due to the limited sample size or to the lack of accurate information about type, intensity, and duration of exercise. Moreover, we could not exclude that physical activity may have an impact on inflammation and/or NAC response without affecting bowel tracts.

Similarly, despite the well-recognized role of excess body weight on BC risk [9] and inflammation [36], in this study, we did not find a correlation between bowel [18]F-FDG uptake and BMI. Nevertheless, we have to point out that our population was mainly composed by normal weight women (only the 3.7% of patients were obese), thus possibly mitigating the effect of this parameter on inflammation. Likewise, the lack of correlation between smoking and [18]F-FDG bowel uptake may be due to the small proportion of smokers in our population (i.e., 18.3%).

Some limitations of the present study should be mentioned. First of all, the monocentric design of the study impacted on the sample size. Due to the pilot nature of this study, we did not calculate an a priori formal sample size estimation, so the conclusions drawn by such a small group of patients should be taken with caution. However, this study could provide us the effect size needed to plan a larger observational study to confirm and validate our findings. Furthermore, we recognize that the present study lacks comparison of dietary diversity as a consequence of the small sample size and the omnivorous habits of most patients. On the other hand, we recruited more than 80 patients, which is on average, more than usual for PET studies [51,57,58,59,60,61,62,63,64,65,66]. Moreover, a single center study allows a better uniformity in patients’ recruitment, data collection, and PET scanning procedures. This allowed us to exclude potential interferences in the results. In fact, the observed increased bowel [18]F-FDG uptake could be due to interfering factors different from eating habits. However, all women followed PET preparation guidelines, including at least 6 h fasting and avoiding the consumption of carbohydrates on the evening before in order to minimize variability due to the last meal before PET. In addition, during the analyses, we took into consideration all comorbidities of our population, without finding differences in bowel [18]F-FDG uptake between patients with a history of gastrointestinal diseases and women without. Finally, none of our patients assumed the oral hypoglycemic treatment Metformin that is known to affect intestinal [18]F-FDG uptake in diabetic patients [67]. Only one of the enrolled women was diabetic and she treated it through an insulin pen.

## 5. Conclusions

In conclusion, albeit the pilot nature of the study, the most striking result of our study is to have pinpointed an association between NAC not-complete response and increased levels of colon [18]F-FDG uptake, which are affected by BC patients’ pro-inflammatory eating habits (i.e., consumption of unhealthy foods/drinks), for the first time. Additional investigations in enlarged cohorts are needed to confirm and validate our proof-of-principle study and to deeply investigate whether [18]F-FDG PET/CT could be an easy instrument for identifying BC patients who could be referred for nutritional counseling. Moreover, ongoing studies on transcriptome profiling will enhance our understanding of the interaction between bowel inflammation and NAC response in BC.

## Figures and Tables

**Table 1:** Demographic and clinical BC patients’ characteristics.

	Patients (*n* = 82)	
	*n*	%
**Age**		
<50	46	56.1
50–64	29	35.4
≥65	7	8.5
**Smoke**		
no	53	64.6
yes	15	18.3
former	14	17.1
**Menopause**		
no	47	57.3
yes	28	34.1
peri	7	8.5
**BMI**		
<18.5	4	4.9
18.5–24.9	50	61.0
25–29.9	25	30.5
≥30	3	3.7
**Comorbidies**		
None	54	65.9
Intestinal	8	9.8
Others	17	20.7
Intestinal + others	3	3.7

BC, breast cancer; BMI, body mass index.

**Table 2:** Histopathological characteristics of BC (*n* = 82).

	no pCR (*n* = 45)		pCR (*n* = 37)	
	*n*	%	*n*	%
**Stage**				
IA	2	4.4	6	16.2
IIA	17	37.8	19	51.4
IIB	18	40.0	9	24.3
IIIA	6	13.3	3	8.1
IIIB	1	2.2	0	0
IIIC	1	2.2	0	0
**Grade**				
G1	0	0	0	0
G2	16	35.6	9	24.3
G3	29	64.4	28	75.7
**Subtype**				
Luminal A	1	2.2	0	0
Luminal B	3	6.7	4	10.8
HER2+	27	60.0	18	48.6
TNBC	14	31.1	15	40.5

pCR, pathological Complete Response; HER2+, human epidermal growth factor receptor 2 positive; TNBC, triple-negative breast cancer.

**Table 3:** Median of weekly frequency of patients’ food consumption. Range in the brackets.

	Weekly Frequency Consumption Median
Milk	1 (0–7)
Dairy products	2 (0–7)
Alcoholic drinks	0 (0–7)
Spirits	0 (0–2)
White meat	2 (0–6)
Red meat	1 (0–3)
Eggs	1 (0–5)
Fruit	7 (0–7)
Vegetables	7 (1–7)
Fish	1.5 (0–6)
Pulses	1 (0–7)
Cereals	7 (0–7)
Cured meats	2 (0–5)
Salty snacks	1 (0–7)
Sweet snacks/drinks	2.5 (0–7)
Nuts	1 (0–7)

**Table 4:** Weekly frequencies of patients’ physical activity.

	Patients (*n* = 82)	
	*n*	%
Exercise weekly frequency		
0	37	45.1
1–3	26	31.7
>3	17	20.7
missing	2	2.4

**Table 5:** Pearson’s correlation (r) e relative *p*-values (*p*) between bowel [18]F-FDG uptake and patients’ habits.

	Colon SUV_mean_		Rectum SUV_mean_	
	r	*p*	r	*p*
Pro-inflammatory drinks	+0.33	<0.01	+0.14	0.51
Pro-inflammatory foods	+0.25	0.04	+0.02	0.86
Anti-inflammatory foods	−0.21	0.05	−0.23	0.04
Physical activity	−0.20	0.46	−0.11	0.64
Smoke	−0.30	0.81	−0.07	0.96
BMI	+0.17	0.79	+0.15	0.83

SUV_mean_, mean standardized uptake value; BMI, body mass index.

## Data Availability

The data presented in this study are available on request from the corresponding author. The data are not publicly available due to privacy issue.

## Supplementary Materials

The following supporting information can be downloaded at: https://www.mdpi.com/article/10.3390/nu15010211/s1, Figure S1: Flowchart of the recruitment and follow-up process; Figure S2: The survey fulfilled by the enrolled BC patients before NAC; Table S1: Discriminant Function Analysis (DFA) Summary.

## References

[1] Sung H., Ferlay J., Siegel R.L., Laversanne M., Soerjomataram I., Jemal A., Bray F. (2021). Global Cancer Statistics 2020: GLOBOCAN Estimates of Incidence and Mortality Worldwide for 36 Cancers in 185 Countries. CA Cancer J. Clin..

[2] Caprara G., Tieri M., Fabi A., Guarneri V., Falci C., Dieci M.V., Turazza M., Ballardini B., Bin A., Cinieri S. (2021). Results of the ECHO (Eating habits CHanges in Oncologic patients) Survey: An Italian Cross-Sectional Multicentric Study to Explore Dietary Changes and Dietary Supplement Use; in Breast Cancer Survivors. Front. Oncol..

[3] Chlebowski R.T., Aragaki A.K., Anderson G.L., Pan K., Neuhouser M.L., Manson J.E., Thomson C.A., Mossavar-Rahmani Y., Lane D.S., Johnson K.C. (2020). Dietary Modification and Breast Cancer Mortality: Long-Term Follow-Up of the Women’s Health Initiative Randomized Trial. J. Clin. Oncol..

[4] Ligibel J.A., Bohlke K., May A.M., Clinton S.K., Demark-Wahnefried W., Gilchrist S.C., Irwin M.L., Late M., Mansfield S., Marshall T.F. (2020). Exercise; Diet; and Weight Management During Cancer Treatment: ASCO Guideline. J. Clin. Oncol..

[5] Mili N., Paschou S.A., Goulis D.G., Dimopoulos M.A., Lambrinoudaki I., Psaltopoulou T. (2021). Obesity; metabolic syndrome; and cancer: Pathophysiological and therapeutic associations. Endocrine.

[6] Pedersini R., di Mauro P., Bosio S., Zanini B., Zanini A., Amoroso V., Turla A., Vassalli L., Ardine M., Monteverdi S. (2021). Changes in eating habits and food preferences in breast cancer patients undergoing adjuvant chemotherapy. Sci. Rep..

[7] Steck S.E., Murphy E.A. (2020). Dietary patterns and cancer risk. Nat. Rev. Cancer..

[8] Toledo E., Salas-Salvadó J., Donat-Vargas C., Buil-Cosiales P., Estruch R., Ros E., Corella D., Fitó M., Hu F.B., Arós F. (2015). Mediterranean Diet and Invasive Breast Cancer Risk Among Women at High Cardiovascular Risk in the PREDIMED Trial: A Randomized Clinical Trial. JAMA Intern. Med..

[9] Rock C.L., Thomson C., Gansler T., Gapstur S.M., McCullough M.L., Patel A.V., Andrews K.S., Bandera E.V., Spees C.K., Robien K. (2020). American Cancer Society guideline for diet and physical activity for cancer prevention. CA Cancer J. Clin..

[10] Thomson C.A. (2012). Diet and breast cancer: Understanding risks and benefits. Nutr. Clin. Pract..

[11] Arends J., Bachmann P., Baracos V., Barthelemy N., Bertz H., Bozzetti F., Fearon K., Hütterer E., Isenring E., Kaasa S. (2017). ESPEN guidelines on nutrition in cancer patients. Clin. Nutr..

[12] Carayol M., Ninot G., Senesse P., Bleuse J.P., Gourgou S., Sancho-Garnier H., Sari C., Romieu I., Romieu G., Jacot W. (2019). Short- and long-term impact of adapted physical activity and diet counseling during adjuvant breast cancer therapy: The “APAD1” randomized controlled trial. BMC Cancer..

[13] De Cicco P., Catani M.V., Gasperi V., Sibilano M., Quaglietta M., Savini I. (2019). Nutrition and Breast Cancer: A Literature Review on Prevention; Treatment and Recurrence. Nutrients..

[14] Kwan M.L., Weltzien E., Kushi L.H., Castillo A., Slattery M.L., Caan B.J. (2009). Dietary patterns and breast cancer recurrence and survival among women with early-stage breast cancer. J. Clin. Oncol..

[15] McTiernan A. (2021). Diet and Prognosis in Women with Breast Cancer. Cancer Epidemiol. Biomark. Prev..

[16] Herrmann K., Benz M.R., Krause B.J., Pomykala K.L., Buck A.K., Czernin J. (2011). (18)F-FDG-PET/CT in evaluating response to therapy in solid tumors: Where we are and where we can go. Q. J. Nucl. Med. Mol. Imaging..

[17] Kubota K. (2001). From tumor biology to clinical Pet: A review of positron emission tomography (PET) in oncology. Ann. Nucl. Med..

[18] Paydary K., Seraj S.M., Zadeh M.Z., Emamzadehfard S., Shamchi S.P., Gholami S., Werner T.J., Alavi A. (2019). The Evolving Role of FDG-PET/CT in the Diagnosis; Staging; and Treatment of Breast Cancer. Mol. Imaging Biol..

[19] Holtmann M.H., Uenzen M., Helisch A., Dahmen A., Mudter J., Goetz M., Schreckenberger M., Galle P.R., Bartenstein P., Neurath M.F. (2012). 18F-Fluorodeoxyglucose positron-emission tomography (PET) can be used to assess inflammation non-invasively in Crohn’s disease. Dig. Dis. Sci..

[20] Rubin D.T., Surma B.L., Gavzy S.J., Schnell K.M., Bunnag A.P., Huo D., Appelbaum D.E. (2009). Positron emission tomography (PET) used to image subclinical inflammation associated with ulcerative colitis (UC) in remission. Inflamm. Bowel Dis..

[21] Sena Y., Matsumoto S., Silman C., Otsuka K., Kiyota T. (2020). Physiological 18F-FDG uptake in the normal adult anal canal: Evaluation by PET/CT. Ann. Nucl. Med..

[22] Boellaard R., Delgado-Bolton R., Oyen W.J., Giammarile F., Tatsch K., Eschner W., Verzijlbergen F.J., Barrington S.F., Pike L.C., Weber W.A. (2015). FDG PET/CT: EANM procedure guidelines for tumour imaging: Version 2.0. Eur. J. Nucl. Med. Mol. Imaging.

[23] CREA Centro di Ricerca Alimenti e Nutrizione (2018). Linee Guida Per Una Sana Alimentazione.

[24] De Sanctis R., Agostinetto E., Masci G., Ferraro E., Losurdo A., Viganò A., Antunovic L., Zuradelli M., Torrisi R.M.C., Santoro A. (2018). Predictive Factors of Eribulin Activity in Metastatic Breast Cancer Patients. Oncology.

[25] Viganò A., Savastano E., Petolicchio B., Toscano M., De Sanctis R., Maestrini I., Di Piero V. (2020). A Study of Clinical Features and Risk Factors of Self-Referring Emergency Department Headache Patients: A Comparison with Headache Center Outpatients. Eur. Neurol..

[26] De Sanctis R., Viganò A., Giuliani A., Gronchi A., De Paoli A., Navarria P., Quagliuolo V., Santoro A., Colosimo A. (2018). Unsupervised versus Supervised Identification of Prognostic Factors in Patients with Localized Retroperitoneal Sarcoma: A Data Clustering and Mahalanobis Distance Approach. Biomed. Res. Int..

[27] Alessiani M., Petolicchio B., De Sanctis R., Squitieri M., Di Giambattista R., Puma M., Franzese C., Toscano M., Derchi C.C., Gilliéron E. (2022). A Propensity Score Matching Study on the Effect of OnabotulinumtoxinA Alone versus Short-Term Psychodynamic Psychotherapy Plus Drug-of-Choice as Preventive Therapy in Chronic Migraine: Effects and Predictive Factors. Eur. Neurol..

[28] Clotas C., Serral G., Vidal Garcia E., Puigpinós-Riera R., DAMA Cohort Group (2021). Dietary changes and food habits: Social and clinical determinants in a cohort of women diagnosed with breast cancer in Barcelona (DAMA cohort). Cancer Causes Control.

[29] Ding S., Chi M.M., Scull B.P., Rigby R., Schwerbrock N.M., Magness S., Jobin C., Lund P.K. (2010). High-fat diet: Bacteria interactions promote intestinal inflammation which precedes and correlates with obesity and insulin resistance in mouse. PLoS ONE.

[30] Malesza I.J., Malesza M., Walkowiak J., Mussin N., Walkowiak D., Aringazina R., Bartkowiak-Wieczorek J., Mądry E. (2021). High-Fat; Western-Style Diet; Systemic Inflammation; and Gut Microbiota: A Narrative Review. Cells.

[31] Beam A., Clinger E., Hao L. (2021). Effect of Diet and Dietary Components on the Composition of the Gut Microbiota. Nutrients..

[32] Esmaillzadeh A., Kimiagar M., Mehrabi Y., Azadbakht L., Hu F.B., Willett W.C. (2007). Dietary patterns and markers of systemic inflammation among Iranian women. J. Nutr..

[33] Holt E.M., Steffen L.M., Moran A., Basu S., Steinberger J., Ross J.A., Hong C.P., Sinaiko A.R. (2009). Fruit and vegetable consumption and its relation to markers of inflammation and oxidative stress in adolescents. J. Am. Diet Assoc..

[34] Nanri A., Moore M.A., Kono S. (2007). Impact of C-reactive protein on disease risk and its relation to dietary factors. Asian Pac. J. Cancer Prev..

[35] Watzl B., Kulling S.E., Möseneder J., Barth S.W., Bub A. (2005). A 4-wk intervention with high intake of carotenoid-rich vegetables and fruit reduces plasma C-reactive protein in healthy; nonsmoking men. Am. J. Clin. Nutr..

[36] Galland L. (2010). Diet and inflammation. Nutr. Clin. Pract..

[37] Nettleton J.A., Steffen L.M., Mayer-Davis E.J., Jenny N.S., Jiang R., Herrington D.M., Jacobs D.R. (2006). Dietary patterns are associated with biochemical markers of inflammation and endothelial activation in the Multi-Ethnic Study of Atherosclerosis (MESA). Am. J. Clin. Nutr..

[38] Moasses-Ghafari B., Fallahi B., Esfehani A.F., Eftekhari M., Rahmani K., Eftekhari A., Geramifar P. (2021). Effect of Diet on Physiologic Bowel 18F-FDG Uptake. J. Nucl. Med. Technol..

[39] Fallahi B., Moasses-Ghafari B., Fard-Esfahani A., Geramifar P., Beiki D., Emami-Ardekani A., Eftekhari M. (2017). Factors influencing the pattern and intensity of myocardial 18F-FDG uptake in oncologic PET-CT imaging. Iran J. Nucl. Med..

[40] Cortazar P., Zhang L., Untch M., Mehta K., Costantino J.P., Wolmark N., Bonnefoi H., Cameron D., Gianni L., Valagussa P. (2014). Pathological complete response and long-term clinical benefit in breast cancer: The CTNeoBC pooled analysis. Lancet.

[41] Khalili H., Chan S.S.M., Lochhead P., Ananthakrishnan A.N., Hart A.R., Chan A.T. (2018). The role of diet in the aetiopathogenesis of inflammatory bowel disease. Nat. Rev. Gastroenterol. Hepatol..

[42] Marsh A., Radford-Smith G., Banks M., Lord A., Chachay V. (2022). Dietary intake of patients with inflammatory bowel disease aligns poorly with traditional Mediterranean diet principles. Nutr. Diet..

[43] Ioniță-Mîndrican C.B., Ziani K., Mititelu M., Oprea E., Neacșu S.M., Moroșan E., Dumitrescu D.E., Roșca A.C., Drăgănescu D., Negrei C. (2022). Therapeutic Benefits and Dietary Restrictions of Fiber Intake: A State of the Art Review. Nutrients.

[44] Illescas O., Rodríguez-Sosa M., Gariboldi M. (2021). Mediterranean Diet to Prevent the Development of Colon Diseases: A Meta-Analysis of Gut Microbiota Studies. Nutrients.

[45] Vernia F., Longo S., Stefanelli G., Viscido A., Latella G. (2021). Dietary Factors Modulating Colorectal Carcinogenesis. Nutrients.

[46] Campaniello D., Corbo M.R., Sinigaglia M., Speranza B., Racioppo A., Altieri C., Bevilacqua A. (2022). How Diet and Physical Activity Modulate Gut Microbiota: Evidence, and Perspectives. Nutrients.

[47] Lozupone C.A., Stombaugh J.I., Gordon J.I., Jansson J.K., Knight R. (2012). Diversity; stability and resilience of the human gut microbiota. Nature.

[48] Abedpoor N., Taghian F., Hajibabaie F. (2022). Physical activity ameliorates the function of organs via adipose tissue in metabolic diseases. Acta Histochem..

[49] Hajibabaie F., Abedpoor N., Assareh N., Tabatabaiefar M.A., Shariati L., Zarrabi A. (2022). The Importance of SNPs at miRNA Binding Sites as Biomarkers of Gastric and Colorectal Cancers: A Systematic Review. J. Pers. Med..

[50] Abedpoor N., Taghian F., Hajibabaie F. (2022). Cross Brain-Gut Analysis Highlighted Hub Genes and LncRNA Networks Differentially Modified During Leucine Consumption and Endurance Exercise in Mice with Depression-Like Behaviors. Mol. Neurobiol..

[51] Kang J.Y., Kim H.N., Chang Y., Yun Y., Ryu S., Shin H., Kim H.L. (2017). Gut microbiota and physiologic bowel 18F-FDG uptake. EJNMMI Res..

[52] Yoon H.J., Kim H.N., Bang J.I., Lim W., Moon B.I., Paik N.S., Kim B.S., Kim H.L. (2019). Physiologic intestinal 18F-FDG uptake is associated with alteration of gut microbiota and proinflammatory cytokine levels in breast cancer. Sci. Rep..

[53] Kumral D., Zfass A.M. (2018). Gut Movements: A Review of the Physiology of Gastrointestinal Transit. Dig. Dis. Sci..

[54] Barone M., D’Amico F., Brigidi P., Turroni S. (2022). Gut microbiome-micronutrient interaction: The key to controlling the bioavailability of minerals and vitamins?. Biofactors.

[55] Barleben A., Mills S. (2010). Anorectal anatomy and physiology. Surg. Clin. N. Am..

[56] Angulo J., El Assar M., Álvarez-Bustos A., Rodríguez-Mañas L. (2020). Physical activity and exercise: Strategies to manage frailty. Redox Biol..

[57] Basu S., Chen W., Tchou J., Mavi A., Cermik T., Czerniecki B., Schnall M., Alavi A. (2008). Comparison of triple-negative and estrogen receptor-positive/progesterone receptor-positive/HER2-negative breast carcinoma using quantitative fluorine-18 fluorodeoxyglucose/positron emission tomography imaging parameters: A potentially useful method for disease characterization. Cancer.

[58] Williams J.M., Rani S.D., Li X., Arlinghaus L.R., Lee T.C., MacDonald L.R., Partridge S.C., Kang H., Whisenant J.G., Abramson R.G. (2015). Comparison of prone versus supine 18F-FDG-PET of locally advanced breast cancer: Phantom and preliminary clinical studies. Med. Phys..

[59] Jeong Y., Baek S., Park J.W., Joo J.H., Kim J.S., Lee S.W. (2017). Lymph node standardized uptake values at pre-treatment 18F-fluorodeoxyglucose positron emission tomography as a valuable prognostic factor for distant metastasis in nasopharyngeal carcinoma. Br. J. Radiol..

[60] Husi K., Pinczés L.I., Fejes Z., Nagy B., Illés Á., Miltényi Z. (2022). Combined prognostic role of TARC and interim 18F-FDG PET/CT in patients with Hodgkin lymphoma-real world observational study. Hell. J. Nucl. Med..

[61] Guzmán Ortiz S., Mucientes Rasilla J., Vargas Núñez J.A., Royuela A., Rodríguez Carrillo J.L., Dotor de Lama A., Navarro Matilla M.B., Mitjavila Casanovas M. (2022). Evaluation of the prognostic value of the metabolic volumetric parameters calculated with 18F-FDG PET/CT and its value added to the molecular characteristics in patients with diffuse large B-cell lymphoma. Rev. Esp. Med. Nucl. Imagen. Mol..

[62] Wu J., Deng H., Zhong H., Wang T., Rao Z., Wang Y., Chen Y., Zhang C. (2022). Comparison of 68Ga-FAPI and 18F-FDG PET/CT in the Evaluation of Patients With Newly Diagnosed Non-Small Cell Lung Cancer. Front. Oncol..

[63] Guo R., Xu P., Cheng S., Lin M., Zhong H., Li W., Huang H., Ouyang B., Yi H., Chen J. (2020). Comparison of Nasopharyngeal MR; 18 F-FDG PET/CT; and 18 F-FDG PET/MR for Local Detection of Natural Killer/T-Cell Lymphoma; Nasal Type. Front. Oncol..

[64] Kampe K.K., Rotermund R., Tienken M., Thomalla G., Regier M., Klutmann S., Kluge S. (2017). Diagnostic Value of Positron Emission Tomography Combined with Computed Tomography for Evaluating Critically Ill Neurological Patients. Front. Neurol..

[65] Bailly C., Eugène T., Couec M.L., Strullu M., Frampas E., Campion L., Kraeber-Bodéré F., Bodet-Milin C. (2014). Prognostic Value and Clinical Impact of (18)FDG-PET in the Management of Children with Burkitt Lymphoma after Induction Chemotherapy. Front. Med..

[66] Lu X.R., Qu M.M., Zhai Y.N., Feng W., Gao Y., Lei J.Q. (2021). Diagnostic role of 18F-FDG PET/MRI in the TNM staging of breast cancer: A systematic review and meta-analysis. Ann. Palliat. Med..

[67] Yoon H.J., Kim H.N., Yun Y., Kim Y., Ha A.N., Kim H.L., Kim B.S. (2015). Background Intestinal 18F-FDG Uptake Is Related to Serum Lipid Profile and Obesity in Breast Cancer Patients. PLoS ONE.

