# Supplementary material for: The Relationship among Bowel [18]F-FDG PET Uptake, Pathological Complete Response, and Eating Habits in Breast Cancer Patients Undergoing Neoadjuvant Chemotherapy
Source: Nutrients. 2023 Jan 1;15(1):211. doi: 10.3390/nu15010211 (PMC9824388; doi:10.3390/nu15010211)
Supplement: Supplementary file 1 [file nutrients-15-00211-s001.zip › Figure S1.pdf]

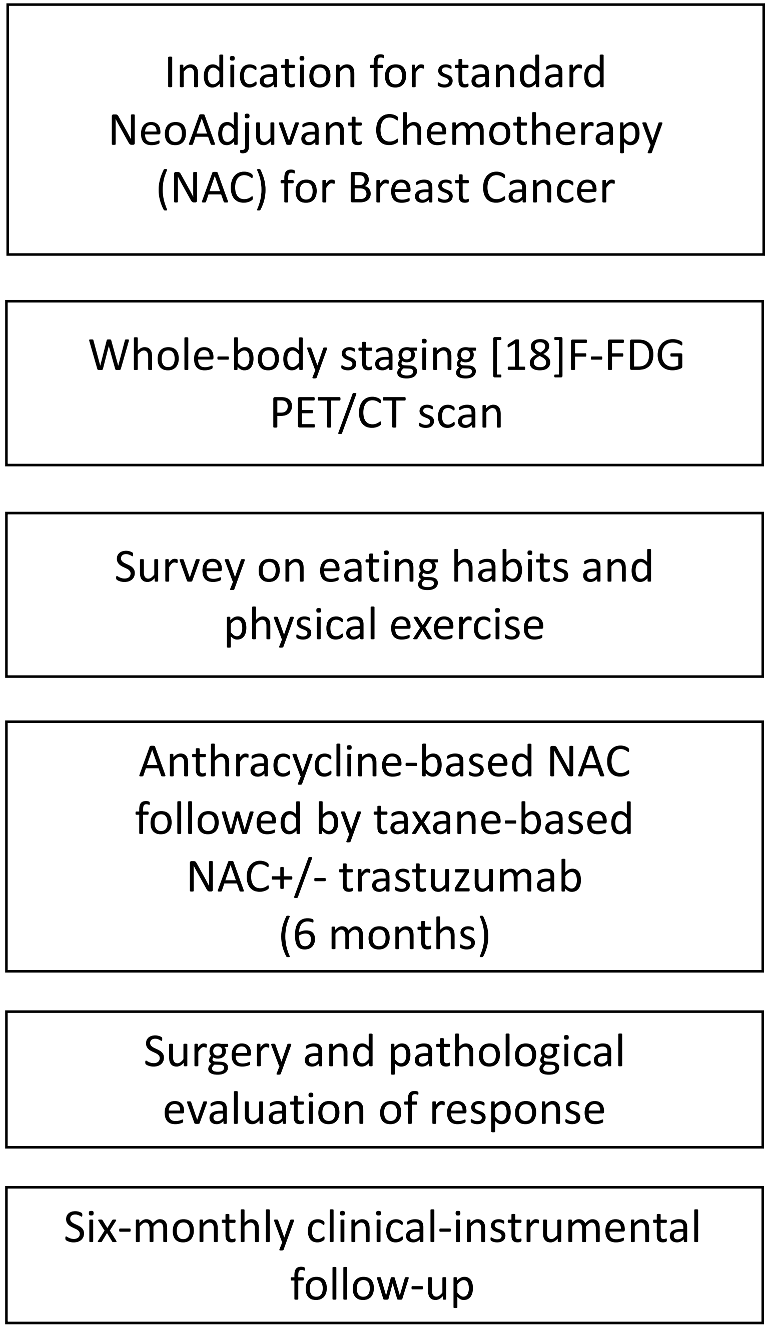
Indication for standard NeoAdjuvant Chemotherapy (NAC) for Breast Cancer
Whole-body staging ^{18}F-FDG PET/CT scan
Survey on eating habits and physical exercise
Anthracycline-based NAC followed by taxane-based NAC+/- trastuzumab (6 months)
Surgery and pathological evaluation of response
Six-monthly clinical-instrumental follow-up
