# Supplementary material for: The Relationship among Bowel [18]F-FDG PET Uptake, Pathological Complete Response, and Eating Habits in Breast Cancer Patients Undergoing Neoadjuvant Chemotherapy
Source: Nutrients. 2023 Jan 1;15(1):211. doi: 10.3390/nu15010211 (PMC9824388; doi:10.3390/nu15010211)
Supplement: Supplementary file 1 [file nutrients-15-00211-s001.zip › Figure S2.pdf]

Name____________________ Surname_________________ Date________________________

1. *How do you define your diet?*

□ omnivorous or varied □ vegetarian □ vegan

2. *How many <u>times a week</u> do you eat:*

milk _____

dairy products _____

alcohol _____

spirits _____

white meat _____

red meat _____

eggs _____

fruits _____; average number of portions per day _________

vegetables _____; average number of portions per day _________

fish _____

legumes _____

cereals (e.g., pasta, rice, bread) ______

gluten: yes / no

integral: yes / no

cured meat _______

salty snacks ________

sweet snacks and sweet drinks __________

dried fruit _______

3. *Did you choose this kind of diet following counselling by:*

□ nutritionist

□ oncologist

□ breast oncologist

□ other specialist

□ family doctor

□ other professional figure

□ other figure

4. *Did you take antibiotics during the last month?* □ yes □ no

If yes, which one and how long? ____

5. *Did you travel abroad during the last 3 months?* □ yes □ no

If yes, where?

____

6. *Did you ever suffer of parasitosis?* □ yes □ no

If yes, which kind?

____

7. *How do you define your faeces?* □ liquid □ soft □ hard

8. *Check the picture that most frequently reflects your daily stool:*

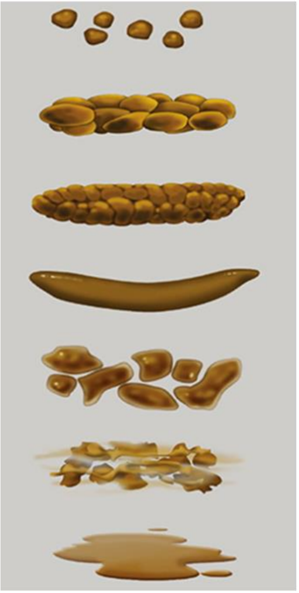

□

□

□

□

□

□

□

9. *How many weekly bowel movements do you have?*
____

10. *Do you suffer from intestinal disorders?* □ yes □ no
If yes, which ones and how often? ____

11. *How many times a week do you exercise?* ________; *what kind of physical activity do you do?*
____
