# Supplementary material for: The Relationship among Bowel [18]F-FDG PET Uptake, Pathological Complete Response, and Eating Habits in Breast Cancer Patients Undergoing Neoadjuvant Chemotherapy
Source: Nutrients. 2023 Jan 1;15(1):211. doi: 10.3390/nu15010211 (PMC9824388; doi:10.3390/nu15010211)
Supplement: Supplementary file 1 [file nutrients-15-00211-s001.zip › Table S1.pdf]

Table S1. Discriminant Function Analysis (DFA) Summary.

	**Wilks' - Lambda**	**Partial - Lambda**	**F-remove - (1.67)**	**p-level**	**Toler.**	**1-Toler. - (R-Sqr.)**
**Colon SUV_{mean}**	0.918325	0.890059	8.275905	0.005384	0.386007	0.613993
**Cured meat**	0.897467	0.910745	6.566163	0.012647	0.635799	0.364201
**Rectum SUV_{mean}**	0.860525	0.949843	3.537979	0.064325	0.422715	0.577285
**Fruits**	0.833850	0.980228	1.351428	0.249153	0.916603	0.083397
**Alcoholic drinks**	0.835096	0.978766	1.453526	0.232204	0.818643	0.181357
**Red meat**	0.832569	0.981736	1.246431	0.268223	0.674051	0.325950
